# Nicotine Dependence and Its Risk Factors Among Users of Veterans Health Services, 2008-2009

**Published:** 2011-10-15

**Authors:** Jack Tsai, Ellen L. Edens, Robert A. Rosenheck

**Affiliations:** VA New England Mental Illness Research, Education, and Clinical Center. Dr Tsai is also affiliated with the VA Connecticut Healthcare System, West Haven, Connecticut, and the Department of Psychiatry, Yale University, New Haven, Connecticut.; VA Connecticut Healthcare System, West Haven, Connecticut. Dr Edens is also affiliated with the Department of Psychiatry, Yale University, New Haven, Connecticut; VA New England Mental Illness Research, Education, and Clinical Center, West Haven, Connecticut. Dr Rosenheck is also affiliated with the VA Connecticut Healthcare System, West Haven, Connecticut, the Department of Psychiatry, Yale University, New Haven, Connecticut, and the Yale School of Public Health, New Haven, Connecticut

## Abstract

**Introduction:**

Tobacco use is the leading preventable cause of death in the United States and is disproportionately higher among veterans than nonveterans. We examined the prevalence of nicotine dependence and its associated risk factors among veterans who used health services in the US Department of Veterans Affairs (VA) system.

**Methods:**

Using a case-control design, we compared all VA health service users in fiscal year 2008-2009 (N = 5,031,381) who received a nicotine dependence diagnosis with those who did not. Independent risk and protective factors associated with receiving a nicotine dependence diagnosis were identified using logistic regression analysis. We conducted subgroup analyses on 2 groups of particular policy concern: homeless veterans and veterans who served in Iraq and Afghanistan.

**Results:**

Among all recent VA health service users, 15% (n = 749,353) received a diagnosis of nicotine dependence. Substance abuse, other mental health diagnoses, and homelessness were identified as major risk factors. Veterans who served in Iraq and Afghanistan were not found to be at increased risk compared to veterans from other war eras. Major risk and protective factors within the subgroups of homeless veterans and veterans who served in Iraq and Afghanistan were broadly similar to those in the general VA population.

**Conclusion:**

Given that other studies have found higher rates of nicotine dependence among veterans, this risk behavior may be underdiagnosed in VA medical records. Veterans who are homeless or have mental health or substance abuse problems are at highest risk and should be targeted for smoking prevention and cessation interventions. These results support, in principle, efforts to integrate smoking cessation programs with mental health and homeless services.

## Introduction

Tobacco use is the leading preventable cause of illness, disability, and premature death in the United States (1). Smoking is responsible for 1 in 5 deaths, resulting in approximately 443,000 avoidable deaths per year (2). These rates are disproportionately higher among veterans because both active-duty personnel and veterans are more likely to have ever smoked or to currently smoke than the adult civilian nonveteran population (3,4). Thus, Department of Veteran Affairs (VA) provides smoking cessation interventions and programs to its health system users (5,6). However, most veterans who smoke and use VA health services report they do not receive tobacco cessation treatment (7). Identifying veteran characteristics related to tobacco use can clarify who is most likely to benefit from smoking prevention and cessation interventions and may enhance VA efforts to reduce smoking and smoking-related illnesses.

A nicotine dependence diagnosis is given to people who use tobacco regularly and have become chronically dependent on nicotine. Epidemiological studies have found a 13% point prevalence (8) and 24% lifetime prevalence (9) for nicotine dependence in the general US adult population. Two recent studies found that 26% to 27% of veterans smoke (10,11). No published research study could be found on the prevalence of diagnosed nicotine dependence among VA service users; thus, the extent to which VA clinicians are assessing and documenting nicotine use and dependence is unclear.

Factors related to smoking cessation have been widely studied; preventing nicotine dependence and identifying predictors of it, less so. Tobacco use is more prevalent and intense among psychiatric populations than the overall population. Up to 41% of adults with mental illness smoke (12-14). These adults may be particularly susceptible to nicotine addiction because tobacco positively influences mood (15). Many people who abuse other substances also smoke, and an especially strong correlation has been found between smoking and alcohol abuse (13). However, this association has not been fully investigated in large studies of veterans.

Research is inadequate on nicotine dependence in 2 groups of particular interest to the VA health system: homeless veterans and veterans who served in Iraq and Afghanistan in Operation Iraqi Freedom and Operation Enduring Freedom (OIF/OEF). Homelessness among veterans has been a national problem for more than 2 decades (16-19), and recently interest has been renewed in ending veterans' homelessness and providing all necessary health care interventions to this population (20). As the United States continues to wage war in the Middle East, health care providers have been especially concerned about OIF/OEF veterans who served in Iraq and Afghanistan, who are at risk of developing various physical and mental health problems postdeployment and after military discharge (21-24). Some studies suggest higher rates of smoking among these veterans (6). Because both of these groups are priorities for VA health services, identifying factors related to nicotine dependence in these 2 groups may help target prevention efforts and curb development of smoking-related illnesses.

The objective of this study was to examine all recent users of VA health services, a group readily available for smoking prevention and cessation interventions, to identify the prevalence of nicotine dependence diagnoses and determine the risk factors associated with receiving such a diagnosis. A secondary objective was to examine risk factors for nicotine dependence among homeless veterans and OEF/OIF veterans.

## Methods

### Study design

Using a cross-sectional case-control study design, we analyzed VA administrative data for all veterans who used VA health services in fiscal year (FY) 2009 (October 1, 2008, to September 30, 2009) to retrospectively compare veterans who had a nicotine dependence diagnosis to those who did not. We compared groups of veterans on the basis of the following characteristics: sociodemographics, homeless status, OEF/OIF status, use of mental health services, urban/rural residence, income, disability status, and mental health diagnoses. We conducted secondary analyses on homeless veterans and OIF/OEF veterans to identify risk factors among these 2 groups. A nicotine dependence diagnosis, not nicotine dependence per se, was the outcome variable in analyses.

### Sample

The total sample consisted of 5,031,381 veterans who used VA health services during FY 2009. We identified nicotine dependence if the veteran received an *International Classification of Diseases, 9th Revision, Clinical Modification* (ICD-9-CM) (www.cdc.gov/nchs/icd9.htm) diagnostic code of 305.1 during FY 2009, as documented in national administrative files.

We defined homeless veterans as veterans who received either specialized VA homeless services or an ICD-9-CM V60.0 diagnostic code (indicating lack of housing) during FY 2009. We identified OIF/OEF veterans through a file provided to the VA by the Department of Defense.

### Measures

Sociodemographic characteristics included sex, age, race/ethnicity, annual household income, and urban/rural residence. We used the working clinical diagnoses of VA clinicians as recorded in the electronic medical record, and we clustered them together in our analysis as dementia, schizophrenia, major depression, bipolar disorder, posttraumatic stress disorder (PTSD), any anxiety disorder (excluding PTSD), alcohol and other drug use disorders, and any personality disorder. We classified veteran service–connected disability status into 3 groups: not service-connected, service-connected with less than 50% disability, and service-connected with 50% or greater disability. We documented urban/rural status using zip codes and the Rural-Urban Commuting Area Codes developed in 1998 at the University of Washington (25), which allowed us to identify veterans residing in large urban areas, midsize communities, small communities, or isolated rural communities.

### Data analysis

In bivariate comparisons of veterans with a nicotine dependence diagnosis and veterans without the diaganosis, we tested the significance of group differences using χ^2^ tests and calculated odds ratios with 95% confidence intervals. Subsequently, we used logistic regression to identify risk factors and protective factors independently associated with nicotine dependence. We dummy coded variables representing race/ethnicity, urban/rural residence, service-connected disability status, and annual income, with reference categories representing other race/ethnicity, urban location, non–service connected, and incomes less than $7,000, respectively. We conducted subgroup analyses on homeless veterans and OIF/OEF veterans. Again, we used logistic regression to identify risk factors and protective factors independently associated with nicotine dependence within each subgroup. We set the level of significance for all analyses at *P* < .01, and all analyses were performed using SAS for Windows, version 9.2 (SAS Institute, Inc, Cary, North Carolina).

## Results

### Bivariate analyses of all VA health service users

Of all VA health service users in FY 2009, 749,353 (14.9%) received a nicotine dependence diagnosis (Table 1). In bivariate analyses, being male, black, having served in OEF/OIF, being aged 40 to 64 years, having an annual household income of $7,000 to $24,999, being service connected, and living in a rural area were significantly associated with nicotine dependence. Homeless veterans were almost 4 times as likely to receive a nicotine dependence diagnosis as veterans who were not homeless, and veterans who used mental health services were 2.5 times as likely to receive a nicotine dependence diagnosis than were veterans who did not use mental health services. Among mental health service users, 25.5% had a diagnosis of nicotine dependence.

The only protective factor among mental health diagnoses was having a diagnosis of dementia. Veterans who received any other mental health diagnoses (including schizophrenia, affective disorders, anxiety disorders, substance use disorders, and personality disorders) were significantly more likely to have a nicotine dependence diagnosis also. At greatest risk were veterans diagnosed with schizophrenia, an alcohol use disorder, a drug use disorder, or a personality disorder.

### Multivariate analyses

**All VA health service users**

After controlling for other factors, veterans who were male, homeless, black, living in rural areas, using mental health services, and had an annual income of more than $7,000 were at increased risk for a nicotine dependence diagnosis independent of other factors (Table 2). OEF/OIF status, age, and being service-connected were found to be protective factors in this analysis. Again, dementia diagnosis was a protective factor, while all the other mental health diagnoses were risk factors, except that having a personality disorder was no longer significant. In particular, veterans who had an alcohol use disorder were more than 3 times as likely as veterans who did not to also have a nicotine dependence diagnosis.

**Homeless veterans**

We identified 120,234 (2.4%) homeless veterans. Among them, 47,252 (39.3%) received a diagnosis of nicotine dependence. Being male, living in a small or large rural area, having an income of $7,000 to $14,999, and being service-connected with less than 50% disability were significantly predictive of a nicotine dependence diagnosis (Table 3). As in the analysis of all VA health service users, having a diagnosis of dementia was a protective factor among homeless veterans, whereas having any other mental health diagnosis (except personality disorder) was a significant risk factor, particularly alcohol use disorder.

**OEF/OIF veterans**

Of the 200,300 (4.0%) veterans who served in OEF/OIF, 30,297 (15.1%) received a diagnosis of nicotine dependence. Among OEF/OIF veterans, being male, homeless, and younger, living in a rural area, having income of $7,000 to $24,999, and using mental health services were significantly predictive of a nicotine dependence diagnosis (Table 3). OEF/OIF veterans who had a diagnosis of bipolar disorder, anxiety disorder, PTSD, alcohol use disorder, or drug use disorder were also at risk for nicotine dependence.

## Discussion

We found that 15% of all veterans who used VA health services in FY 2009 received a diagnosis of nicotine dependence. Because we analyzed administrative data, we likely underestimated how many veterans actually have nicotine dependence; recent estimates indicate that 26% to 27% of veterans smoke (10,11). Although no previous study to our knowledge has examined the prevalence of nicotine dependence in the population of veterans using health services, our finding suggests nicotine dependence may be underdiagnosed and not adequately documented in VA administrative records. Because smoking is a leading cause of many chronic diseases and deaths (1), it may be beneficial for VA clinicians to better document nicotine use. The benefit of better documentation assumes that assessment and diagnosis lead to increased likelihood of successful intervention; various smoking cessation interventions are effective for veterans (5,26,27).

In identifying major risk factors, veterans who had mental health or substance use disorders were at significantly higher risk of receiving a nicotine dependence diagnosis than veterans who did not have such diagnoses. Among VA mental health service users, one-fourth had a nicotine dependence diagnosis. This result is consistent with previous findings of increased rates of nicotine use among adults with mental illness or substance use disorders in the general population (12-14).

Having an alcohol use disorder was the strongest independent predictor of a nicotine dependence diagnosis, followed closely by a drug use disorder. Veterans who had an alcohol use disorder were more than 3 times as likely and veterans with a drug use disorder were almost 2 times as likely to receive a nicotine dependence diagnosis compared to veterans without such disorders and controlling for other influential factors. VA clinicians may need to pay particular attention to smoking behaviors among veterans with mental illness or substance use disorders, especially because nicotine dependence disproportionately reduces the quality and length of life of people with these disorders in the general population (28). Providing smoking prevention and cessation interventions with other substance abuse and mental health treatment for veterans may be useful; efforts to integrate nicotine cessation programs into VA mental health services have shown some success (5).

Homeless veterans were also at increased risk for nicotine dependence diagnosis (39%), independent of their increased risk for addictive disorders. This finding is consistent with recent studies, which have found that 69% to 73% of homeless people in the general population smoke (29,30). Interestingly, these studies also found that more than one-third of homeless smokers expressed a readiness to quit and more than half received advice to quit from their health care providers, but they were still less likely to quit compared to others in the general population. People with multiple episodes of homelessness were less likely to quit (29). Besides alcohol and drug use as factors associated with smoking in the homeless population, studies have also found out-of-home placement in childhood, victimization while homeless, and smoking intiation at an earlier age are significant factors (29,31). There has been little development of smoking prevention and cessation programs for homeless people, let alone homeless veterans, and more research is needed in this area.

OEF/OIF status was protective against nicotine dependence diagnosis, in contrast to previous studies, which relied on self-report (6). It is worth reiterating that we did not examine nicotine dependence, per se, but rather how often it was diagnosed, which may explain the difference in findings and suggests nicotine dependence is not adequately assessed among OEF/OIF veterans, who are likely seeking treatment for more pressing health issues. We found that 15% of OEF/OIF veterans who received VA health services in FY 2009 received a nicotine dependence diagnosis. Substance use disorders were still significant risk factors, but OEF/OIF status alone did not increase risk for a nicotine dependence diagnosis. Among both OEF/OIF and homeless veterans, we consistently found that veterans who were male, low-income, and living in a rural area were at higher risk of receiving a nicotine dependence diagnosis. Dementia was found to be a protective factor, which may be because of its effects on general life functioning and behaviors, including smoking.

This study has several limitations. Administrative records are not always complete or reliable. VA clinicians may have neglected to document nicotine dependence in the face of presenting primary diagnoses, which only illustrates the importance for VA clinicians to conduct comprehensive assessments of patients that include questions about smoking behaviors. We focused on identifying risk factors of a clinical diagnosis of nicotine dependence, which may be different from factors related to actual nicotine dependence. There may also be other correlates of nicotine dependence that we did not address in our analyses, such as certain medical conditions and unmeasured individual characteristics. Given our large sample size, analyses were sensitive to statistical significance, so we focused on odds ratios to identify major risk factors. Although we identified some correlates for nicotine dependence among veterans, we could not examine the causal pathways through which these factors increase risk because our data were cross-sectional. Future research and development of assessment, documentation, and interventions in this area are needed.

Our results suggest veterans are underdiagnosed for nicotine dependence and that better assessment and documentation methods are needed in the VA health system. Veterans who are homeless, have a mental illness, or have a substance use disorder may be particularly vulnerable to dependence on nicotine, and targeted outreach and intervention for these groups may be needed. This study may contribute to improved targeting of smokng prevention and cessation efforts in the VA health care system.

## Figures and Tables

**Table 1:** Bivariate Analysis of Demographic Characteristics, Health Service Use, and Mental Health Diagnoses With Nicotine Dependence Among Veterans,^a^ 2008-2009

**Characteristic/Use/Diagnosis**	All VA Service Users, n (%) (N = 5,031,381)	VA Service Users With Nicotine Dependence, n (%) (n = 749,353)	Likelihood of Being Diagnosed With Nicotine Dependence, OR (95% CI)^b^
**Sex**			
Male	4,745,729 (94.3)	708,256 (14.9)	1.0 (1.0-1.1)
Female	285,652 (5.7)	41,097 (14.4)	1 [Reference]
**Veteran status**			
OIF/OEF	200,300 (4.0)	30,297 (15.1)	1.0 (1.0-1.0)
Other war eras	4,831,081 (96.0)	719,056 (14.9)	1 [Reference]
**Homeless status**			
Homeless	120,234 (2.4)	47,252 (39.3)	3.9 (3.8-3.9)
Not homeless	4,911,147 (97.6)	702,101 (14.3)	1 [Reference]
**Age, y^b^**			
<40	548,827 (10.0)	77,549 (14.1)	0.9 (0.9-0.9)
Not <40	4,482,554 (89.1)	671,804 (15.0)	1 [Reference]
40-49	474,444 (9.4)	99,805 (21.0)	1.6 (1.6-1.6)
Not 40-49	4,556,937 (90.6)	649,548 (14.3)	1 [Reference]
50-64	1,855,142 (36.9)	417,610 (22.5)	2.5 (2.5-2.5)
Not 50-64	3,176,239 (63.1)	331,743 (10.4)	1 [Reference]
65-74	931,971 (18.5)	107,565 (11.5)	0.7 (0.7-0.7)
Not 65-74	4,099,410 (81.6)	641,788 (15.7)	1 [Reference]
75-85	975,536 (19.4)	42,806 (4.4)	0.2 (0.2-0.2)
Not 75-85	4,055,845 (80.6)	706,547 (17.4)	1 [Reference]
>85	245,461 (4.9)	4018 (1.6)	0.1 (0.1-0.1)
Not >85	4,785,920 (95.1)	745,335 (15.6)	1 [Reference]
**Race/ethnicity**			
White/unknown	4,667,988 (92.8)	683,919 (14.6)	0.8 (0.8-0.8)
Not white/unknown	363,393 (7.2)	65,434 (18.0)	1 [Reference]
Black	269,618 (5.4)	54,278 (20.1)	1.5 (1.5-1.5)
Not black	4,761,763 (94.6)	695,075 (14.6)	1 [Reference]
Hispanic	101,633 (2.0)	12,271 (12.1)	0.8 (0.8-0.8)
Not Hispanic	4,929,748 (98.0)	737,082 (15.0)	1 [Reference]
**Urban/rural residence**			
Urban	3,403,266 (70.0)	492,611 (14.5)	0.7 (0.7-0.8)
Not urban	1,456,514 (28.9)	235,919 (16.2)	1 [Reference]
Large rural	596,785 (12.3)	96,631 (16.2)	1.3 (1.3-1.3)
Not large rural	4,755,606 (94.5)	631,899 (13.3)	1 [Reference]
Small rural	479,733 (9.9)	77,951 (16.2	1.3 (1.2-1.3)
Not small rural	4,872,658 (96.8)	650,579 (13.4)	1 [Reference]
Isolated rural	379,996 (7.8)	61,337 (16.1)	1.2 (1.2-1.3)
Not isolated rural	4,972,395 (98.8)	667,193 (13.4)	1 [Reference]
**Annual income, $**			
<7,000	1,684,080 (33.5)	224,110 (13.3)	0.8 (0.8-0.8)
Not <7,000	3,347,301 (66.5)	525,243 (15.7)	1 [Reference]
7,000-14,999	863,429 (17.2)	174,188 (20.2)	1.6 (1.6-1.6)
Not 7,000-14,999	4,167,952 (82.8)	575,165 (13.8)	1 [Reference]
15,000-24,999	620,426 (12.3)	101,087 (16.3)	1.1 (1.1-1.1)
Not 15,000-24,999	4,410,955 (87.7)	648,266 (14.7)	1 [Reference]
≥25,000	1,863,446 (37.0)	249,968 (13.4)	0.8 (0.8-0.8)
Not ≥25,000	3,167,935 (63.0)	499,385 (15.8)	1 [Reference]
**Disability status**			
Not service-connected	3,212,820 (63.8)	479,899 (14.9)	0.9 (0.9-0.9)
Service-connected	1,818,561 (36.1)	269,454 (14.8)	1 [Reference]
Service-connected, <50% disabled	943,456 (18.8)	128,361 (13.6)	1.0 (1.0-1.0)
Not service-connected, <50% disabled	4,567,824 (90.8)	620,992 (13.6)	1 [Reference]
Service-connected, ≥50% disabled	875,105 (17.4)	141,093 (16.1)	1.3 (1.3-1.3)
Not service-connected, ≥50% disabled	4,636,175 (92.1)	608,260 (13.1)	1 [Reference]
**Mental health service use**			
Any	1,102,846 (21.9)	281,266 (25.5)	2.5 (2.5-2.5)
None	3,928,535 (78.1)	468,087 (11.9)	1 [Reference]
**Mental health diagnosis**			
Dementia	58,157 (1.2)	3,226 (5.6)	0.3 (0.3-0.3)
No dementia	4,973,224 (98.8)	746,127 (15.0)	1 [Reference]
Schizophrenia	91,228 (1.8)	30,916 (33.4)	3.0 (3.0-3.1)
No schizophrenia	4,940,153 (98.2)	718,437 (14.5)	1 [Reference]
Bipolar disorder	102,636 (2.0)	32,608 (31.8)	2.7 (2.7-2.8)
No bipolar disorder	4,928,745 (98.0)	716,745 (14.5)	1 [Reference]
Major depression	251,560 (5.0)	64,732 (25.7)	2.1 (2.1-2.1)
No major depression	4,779,821 (95.0)	684,621 (14.3)	1 [Reference]
Anxiety disorder^c^	365,270 (7.3)	87,406 (23.9)	1.9 (1.9-1.9)
No anxiety disorder	4,666,111 (92.7)	661,947 (14.2)	1 [Reference]
PTSD	494,202 (9.8)	118,495 (24.0)	2.0 (1.9-2.0)
No PTSD	4,537,179	630,858 (13.9)	1 [Reference]
Alcohol use disorder	301,214 (6.0)	138,495 (46.0)	5.7 (5.7-5.8)
No alcohol use disorder	4,730,167 (94.0)	610,858 (12.9)	1 [Reference]
Drug use disorder	196,268 (3.9)	91,249 (46.5)	5.5 (5.5-5.6)
No drug use disorder	4,835,113 (96.1)	658,104 (13.6)	1 [Reference]
Personality disorder	43,176 (0.9)	14,869 (34.4)	3.0 (3.0-3.1)
No personality disorder	4,988,205 (99.1)	734,484 (14.7)	1 [Reference]

Abbreviations: VA, Veterans Affairs; OR, odds ratio; CI, confidence interval; OEF/OIF, Operation Enduring Freedom/Operation Iraqi Freedom; PTSD, post-traumatic stress disorder.

a Among veterans who used the US Department of Veterans Affairs health system.

b OR for age represents odds with every increase of 10 years.

c Excludes PTSD.

**Table 2:** Association of Demographic Characteristics, Health Service Use, and Mental Health Diagnoses With Nicotine Dependence Among Veterans,^a^ 2008-2009

Characteristic/Use/Diagnosis	Likelihood of Nicotine Dependence Diagnosis	

	OR (95% CI)^b^ (n = 749,535)	*P* Value^c^
**Sex**		
Female	1 [Reference]	NA
Male	1.5 (1.5-1.5)	<.001
**Veteran status**		
Other war eras	1 [Reference]	NA
OIF/OEF	0.4 (0.4-0.5)	<.001
**Homeless status**		
Not homeless	1 [Reference]	NA
Homeless	1.2 (1.1-1.2)	<.001
**Age^d^**	0.8 (0.8-0.8)	<.001
**Race/ethnicity**		
Other	1 [Reference]	NA
White/unknown	1.1 (1.0-1.3)	.006
Black	1.3 (1.2-1.4)	<.001
Hispanic	0.8 (0.8-0.9)	<.001
**Urban/rural residence**		
Urban	1 [Reference]	NA
Large rural	1.3 (1.3-1.3)	<.001
Small rural	1.3 (1.3-1.3)	<.001
Isolated rural	1.4 (1.3-1.4)	<.001
**Annual income, $**		
<7,000	1 [Reference]	NA
7,000-14,999	1.5 (1.5-1.6)	<.001
15,000-24,999	1.3 (1.3-1.3)	<.001
≥25,000	1.1 (1.1-1.1)	<.001
**Disability status**		
Not service-connected	1 [Reference]	NA
Service-connected, <50% disabled	0.8 (0.8-0.8)	<.001
Service-connected, ≥50% disabled	0.8 (0.8-0.8)	<.001
**Mental health service use**		
None	1 [Reference]	NA
Any	1.3 (1.3-1.3)	<.001
**Mental health diagnosis**		
Not having the diagnosis	1 [Reference]	NA
Dementia	0.5 (0.5-0.6)	<.001
Schizophrenia	1.8 (1.7-1.8)	<.001
Bipolar disorder	1.2 (1.2-1.2)	<.001
Major depression	1.1 (1.0-1.1)	<.001
Anxiety disorder^e^	1.1 (1.1-1.1)	<.001
PTSD	1.2 (1.2-1.2)	<.001
Alcohol use disorder	3.2 (3.1-3.2)	<.001
Drug use disorder	1.8 (1.8-1.9)	<.001
Personality disorder	1.0 (1.0-1.0)	.03

Abbreviations: OR, odds ratio; CI, confidence interval; OEF/OIF, Operation Enduring Freedom/Operation Iraqi Freedom; NA, not applicable; PTSD, post-traumatic stress disorder.

a Among veterans for whom nicotine dependence diagnosis was documented in administrative records of the US Department of Veterans Affairs (VA) health system.

b Veterans with nicotine dependence represent 14.9% of all VA health system users (N = 5,031,381).

c Calculated by using the χ^2^ test.

d OR for age represents odds with every increase of 10 years.

e Excludes PTSD.

**Table 3:** Association of Demographic Characteristics, Health Service Use, and Mental Health Diagnoses With Nicotine Dependence Among Subpopulations of Veterans (n = 749,353),^a^ 2008-2009

Characteristic/Use/Diagnosis	Likelihood of Nicotine Dependence Diagnosis			

	Homeless Veterans, OR (95% CI) (n = 47,252)^b^	*P* Value^c^	OEF/OIF Veterans, OR (95% CI) (n = 30,297)^d^	*P* Value^c^
**Sex**				
Female	1 [Reference]	NA	1 [Reference]	NA
Male	1.2 (1.1-1.2)	<.001	1.4 (1.3-1.4)	<.001
**Veteran status**				
Other war eras	1 [Reference]	NA	1 [Reference]	NA
OEF/OIF	0.8 (0.7-0.8)	<.001	NA	NC
**Homeless status**				
Not homeless	NA	NC	1 [Reference]	NA
Homeless	NA	NC	1.3 (1.2-1.4)	<.001
**Age^e^**	1.0 (1.0-1.0)	.24	1.0 (1.0-1.0)	<.001
**Race/ethnicity**				
Other	1 [Reference]	NA	1 [Reference]	NA
White/unknown	1.2 (0.9-1.7)	.22	0.6 (0.1-4.5)	.59
Black	1.2 (0.8-1.6)	.41	0.5 (0.1-4.3)	.57
Hispanic	0.9 (0.6-1.2)	.36	0.3 (0.0-2.3)	.25
**Urban/rural residence**				
Urban	1 [Reference]	NA	1 [Reference]	NA
Large rural	1.2 (1.1-1.2)	<.001	1.4 (1.4-1.5)	<.001
Small rural	1.2 (1.1-1.3)	<.001	1.5 (1.5-1.6)	<.001
Isolated rural	1.1 (1.0-1.2)	.01	1.5 (1.5-1.6)	<.001
**Annual income, $**				
<7,000	1 [Reference]	NA	1 [Reference]	NA
7,000-14,999	1.1 (1.1-1.1)	<.001	1.1 (1.1-1.2)	<.001
15,000-24,999	1.0 (1.0-1.1)	.66	1.1 (1.0-1.1)	<.001
≥25,000	1.0 (0.9 (1.0)	.28	1.0 (1.0-1.0)	.99
**Disability status**				
Not service-connected	1 [Reference]	NA	1 [Reference]	NA
Service-connected, <50% disabled	0.9 (0.9-1.0)	.004	1.0 (0.9-1.0)	.004
Service-connected, ≥50% disabled	1.0 (0.9-1.0)	.12	0.9 (0.9-0.9)	<.001
**Mental health service use**				
None	1 [Reference]	NA	1 [Reference]	NA
Any	1.1 (1.0-1.1)	.01	1.3 (1.2-1.3)	<.001
**Mental health diagnosis**				
Not having the diagnosis	1 [Reference]	NA	1 [Reference]	NA
Dementia	0.7 (0.5-0.8)	<.001	0.9 (0.4-1.9)	.77
Schizophrenia	1.3 (1.2-1.3)	<.001	1.2 (1.0-1.4)	.01
Bipolar disorder	1.1 (1.1-1.2)	<.001	1.3 (1.2-1.4)	<.001
Major depression	1.2 (1.1-1.2)	<.001	1.0 (1.0-1.1)	.06
Anxiety disorder^f^	1.1 (1.1-1.2)	<.001	1.3 (1.2-1.3)	<.001
PTSD	1.2 (1.1-1.2)	<.001	1.3 (1.3-1.4)	<.001
Alcohol use disorder	2.1 (2.0-2.1)	<.001	2.3 (2.2-2.4)	<.001
Drug use disorder	1.9 (1.9-2.0)	<.001	2.0 (1.9-2.1)	<.001
Personality disorder	1.1 (1.0-1.1)	.02	1.1 (1.0-1.3)	.01

Abbreviations: OR, odds ratio; CI, confidence interval; OEF/OIF, Operation Enduring Freedom/Operation Iraqi Freedom; NA, not applicable; NC, not calculated; PTSD, post-traumatic stress disorder.

a Among veterans for whom nicotine dependence diagnosis was documented in administrative records of the US Department of Veterans Affairs (VA) health system.

b Represents 39.3% of all homeless VA health system users (n = 120,234).

c Calculated by using the χ^2^ test.

d Represents 15.1% of all OEF/OIF VA health system users (n = 200,300).

e OR for age represents odds with every increase of 10 years.

f Excludes PTSD.
